# Non-catalytic participation of the Pin1 peptidyl-prolyl isomerase domain in target binding

**DOI:** 10.3389/fphys.2013.00018

**Published:** 2013-02-13

**Authors:** Brendan T. Innes, Melanie L. Bailey, Christopher J. Brandl, Brian H. Shilton, David W. Litchfield

**Affiliations:** Department of Biochemistry, Schulich School of Medicine and Dentistry, University of Western OntarioLondon, ON, Canada

**Keywords:** peptidyl-prolyl isomerase, Pin1, phosphorylation-dependent interactions, WW domain, peptidyl-prolyl isomerization

## Abstract

Pin1 is a phosphorylation-dependent peptidyl-prolyl isomerase (PPIase) that has the potential to add an additional level of regulation within protein kinase mediated signaling pathways. Furthermore, there is a mounting body of evidence implicating Pin1 in the emergence of pathological phenotypes in neurodegeneration and cancer through the isomerization of a wide variety of substrates at peptidyl-prolyl bonds where the residue preceding proline is a phosphorylated serine or threonine residue (i.e., pS/T-P motifs). A key step in this regulatory process is the interaction of Pin-1 with its substrates. This is a complex process since Pin1 is composed of two domains, the catalytic PPIase domain, and a type IV WW domain, both of which recognize pS/T-P motifs. The observation that the WW domain exhibits considerably higher binding affinity for pS/T-P motifs has led to predictions that the two domains may have distinct roles in mediating the actions of Pin1 on its substrates. To evaluate the participation of its individual domains in target binding, we performed GST pulldowns to monitor interactions between various forms of Pin1 and mitotic phospho-proteins that revealed two classes of Pin-1 interacting proteins, differing in their requirement for residues within the PPIase domain. From these observations, we consider models for Pin1-substrate interactions and the potential functions of the different classes of Pin1 interacting proteins. We also compare sequences that are recognized by Pin1 within its individual interaction partners to investigate the underlying basis for its different types of interactions.

## Introduction

Pin1 is unique amongst all the peptidyl-prolyl isomerases (PPIases) because it interacts with phosphorylated serine or threonine residues followed by a proline (pS/T-P) (Lu et al., 1996; Ranganathan et al., 1997; Yaffe et al., 1997; Lu and Zhou, 2007). Due to this phospho-specificity, Pin1 has been proposed to be a regulatory timer of various cell signaling processes and is involved in many important cellular functions including growth and proliferation, immune responses, transcription, and apoptosis (Lu et al., 2007; Yeh and Means, 2007; Esnault et al., 2008; Takahashi et al., 2008). Pin1 is also implicated in diseases such as cancer and Alzheimer's disease and is a potential therapeutic target (Lu et al., 2006; Yeh and Means, 2007; Takahashi et al., 2008). In spite of the many functional studies and its therapeutic potential, the molecular mechanisms and physiological roles of Pin1 binding and catalysis are not fully elucidated (Lippens et al., 2007).

Pin1 is a 163 residue, two-domain protein with an N-terminal WW domain and C-terminal catalytic or PPIase domain (Ranganathan et al., 1997; Bayer et al., 2003). Both domains recognize pS/T-P motifs (Shen et al., 1998; Lu et al., 1999b). As the WW domain has been reported to have a ten-fold higher binding affinity for peptides than the PPIase domain *in vitro*, it is thought to act as a protein interaction domain, functioning in protein targeting and enhancing substrate specificity (Lu et al., 1999b; Smet et al., 2005). Nevertheless, the catalytic PPIase domain also exhibits phosphate-directed binding and has structural elements critical for this function (Yaffe et al., 1997). Clearly identified in the original structure (Ranganathan et al., 1997), and confirmed by mutagenesis studies performed previously by Behrsin et al. (2007), the residues K63, R68, and R69 form a positively charged phosphate-binding loop to coordinate the phosphorylated serine or threonine. Further investigations aimed at elucidating a role for phospho-protein binding by the PPIase domain have been hampered by the low affinity binding of the PPIase domain and the difficulty in generating full-length phosphorylated substrates for *in vitro* studies. On this basis, it is not known how the two domains of Pin1 coordinate their activities on full-length substrates; it is also unclear whether all target phospho-proteins interact with the two domains of Pin1 in the same manner.

**Figure 1:**
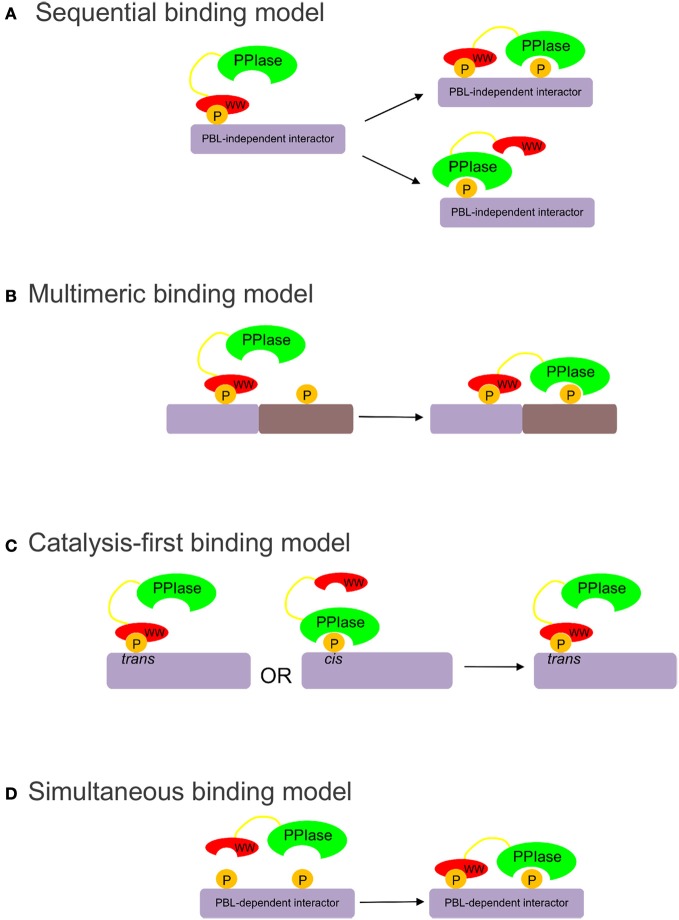
**(A)** The sequential binding model proposes that the WW domain binds first, bringing the PPIase domain proximal to its targets (Zhou et al., 1999; Lu et al., 2002). **(B)** The multimeric binding model proposes that the WW domain anchors Pin1 in multimeric complexes, allowing the PPIase domain to target other substrates in the complex (Jacobs et al., 2003). **(C)** The catalysis-first binding model proposes that the PPIase domain catalyzes the *cis* to *trans* isomerization of the target site to allow *trans*-isomer-specific WW domain binding (Wintjens et al., 2001). **(D)** The simultaneous binding model proposes that the WW and PPIase domains bind simultaneously with low-affinity to multiply phosphorylated targets.

At least three models have been proposed to explain how both domains of Pin1 coordinate binding and isomerization of its substrates: the sequential, multimeric, and catalysis-first models (Figure 1). The sequential model (Figure 1A) is based on the apparent difference in affinity of the two domains for the target sequence. It proposes that the WW domain must either bind first, then release, allowing the PPIase domain to catalyze the isomerization of the binding site; or remain bound, allowing the PPIase domain to act on one or more other sites in the same molecule (Zhou et al., 1999; Lu et al., 2002). This WW-domain-directed sequential model is consistent with the large number of multiply phosphorylated Pin1 substrates. The multimeric model (Figure 1B) proposes that the WW domain anchors Pin1 in multimeric complexes that include the upstream kinase creating the Pin1 binding site (Jacobs et al., 2003). Thus, the substrate is phosphorylated and isomerized by two members of the same complex, with the PPIase domain already in high local concentration when its binding site is created. The catalysis-first model (Figure 1C) proposes that the PPIase domain is required to create WW domain binding sites (Wintjens et al., 2001). In all available structures of the WW-domain bound to substrate peptides, the binding site is in the *trans* conformation (Verdecia et al., 2000; Wintjens et al., 2001; Zhang et al., 2007). If the WW domain exhibited isomer-specific binding, this would give Pin1-catalyzed isomerization a *cis* to *trans* direction. The PPIase domain would isomerize the substrate and form a WW-domain binding site. This would allow the WW-domain to sequester the pool of *trans*-substrate so that the PPIase domain could not catalyze the reverse isomerization.

To elucidate the substrate-binding role of the PPIase domain, wild-type and active site mutants of Pin1 were used to pull down phospho-proteins from mitotically arrested HeLa cell extracts. From these studies we identified a class of Pin1 substrates that require an intact PPIase domain phosphate-binding loop for high affinity binding to full-length Pin1. To explain the binding data, we propose a simultaneous model of binding (Figure 1D), in which certain Pin1 targets containing two or more pS/T-P motifs bind with relatively low affinity to the isolated WW and PPIase domains, but are able to interact simultaneously with both domains to bind full-length Pin1 with high affinity.

## Methods

### Plasmid construction

Constructs for expression of GST, wild-type GST-Pin, and GST-Pin1-C113S have been described previously (Bailey et al., 2008). Human GFP-C1-Cdc25C was a gift from H. Piwnica-Worms and was used as the template for subsequent Cdc25C cloning. Individual WW and PPIase domains of Pin1 were cloned into PCR blunt (Invitrogen) and subcloned using NcoI and HindIII into a pGEX vector. The GST-Pin1-R68A/R69A mutant was generated using the Quikchange II Site-directed mutagenesis kit (Stratagene), according to manufacturer's instructions. pSPORT6-NonO containing human NonO cDNA was purchased from ATCC.

### GST fusion protein purification

GST protein expression and purification was performed as described previously, with bacteria being grown in LB at 37°C and with overnight induction of protein with 0.6 mM isopropylthio-α-D-β-galactoside at 18°C (Bailey et al., 2008). After purification on glutathione-agarose (Sigma) and elution with 10 mM reduced glutathione (Sigma), proteins were dialyzed into PBS with 20% glycerol at 4°C. The protein concentration after dialysis was determined using the Bradford Protein Assay (Bio-Rad) and proteins were stored at −80°C until use.

### Cell culture and transfection

HeLa cells were maintained at 37°C and 5% CO_2_ in DMEM (Invitrogen) supplemented with 10% FBS and 1% penicillin/streptomycin (Invitrogen). Transfection of cells at ~80% confluence was performed using the calcium phosphate method with 80 μg of total DNA/15 cm plate (Olsten et al., 2004). Transfection efficiency was estimated at 50–70% under these conditions. After 16–18 h, cells were washed with phosphate buffered saline (PBS; Invitrogen) and fresh media was added. To arrest cells in mitosis, cells were treated with 0.25 μg/mL nocodazole (Sigma) for 18 h before harvest. Cells were harvested by collecting cells loosely attached to the tissue culture plate and resuspending them in lysis buffer (50 mM Tris-HCl, pH 8, 200 mM NaCl, 10% glycerol, 1% Triton X-100) with added protease and phosphatase inhibitors: 1 mM PMSF, 10 μg/mL pepstatin A 1 mg/mL leupeptin, 1 μM microcystin-LR, 1 mM DTT, 1 mM sodium orthovanadate, and 1 μM okadaic acid. Cells were allowed to lyse on ice on the bench top for 2–3 h and cell debris was then spun down by centrifugation first at 13,000 rpm for 15 min and then at 55,000 rpm for 30 min. Cell lysates were used immediately in pull-downs.

### GST pull-downs

For GST pull-downs, 200 μg of GST fusion protein was bound to glutathione cross-linked to agarose beads (Sigma). Beads were washed twice with PBS buffer and once with lysis buffer then incubated with 0.5–2 mg of HeLa lysate for 1 h at 4°C. Bound proteins were washed with lysis buffer containing the following protease and phosphatase inhibitors: 1 mM PMSF, 10 μg/mL pepstatin A, 1 mg/mL leupeptin, 1 μM microcystin-LR plus 1 mM DTT. After washing 3–5 times, proteins were eluted into SDS sample buffer by boiling. One two-hundredth of the eluted proteins (by volume) of each pull-down was separated, diluted in 20 μL of SDS sample buffer and separated by SDS-PAGE before staining with Coomassie Blue to visualize GST fusion protein loading.

Prominent bands on the stained gel were excised with an Ettan Spot Picker (Amersham) and digested using trypsin at the Functional Proteomics Facility (University of Western Ontario). Samples were run on a MALDI-TOF and identified using the MASCOT program.

### Western blotting

Pull-downs were separated by SDS-PAGE and transferred to polyvinyl difluoride membrane (Millipore). Primary antibodies used include MPM-2 (2 μg/mL; Millipore), Plk1 (1/500; Santa Cruz), Cdc25C (C-20) (1/100; Santa Cruz), SFPQ (1 μg/mL; Abcam), NonO/p54^nrb^ (1 μg/mL; Abcam), HA (12CA5) (1/500; Roche), and gamma tubulin. After incubation with primary antibodies at 4°C overnight, blots were washed and incubated with either GAM or GAR secondary antibodies from Licor Biosciences. Membranes were viewed on a LiCor imager and quantitation was done using Odyssey software (Version 3.0). For quantitation, band intensity on the blot was normalized for the amount of GST fusion protein using the 1/200 Coomassie-stained gel and corrected for the background in the GST lane.

### Binding-site sequence analysis

A comprehensive dataset of Pin1 interactors with defined binding sites was produced from the literature. Only Pin1 targets that had definitive binding sites for Pin1, where mutation or abrogation of the site led to a loss of physical interaction as determined by pull-downs or immunoprecipitations, were used in further analysis. Sequences and general protein data (specifically subcellular location and available structural data) were obtained from the UniProt Knowledgebase (Magrane and the UniProt consortium, 2011). Other data used to characterize the proteins identified (protein function, and roles in cancer and neurodegeneration) were gathered from literature sources, with citations available in Data Sheet 1. Prediction of regions of disorder and secondary structure features were performed using the DISOPRED2 and PSIPRED (Jones, 1999; Ward et al., 2004). Protein kinases acting on the identified sites were predicted using the Group-based Phosphorylation Scoring Method (GPS) tool, but a lack of confidence in the output led to manual annotation of the kinases involved from literature sources, when available (Zhou et al., 2004; Xue et al., 2005). Electrostatic analysis of the Pin1 target sequences was performed using the ExPASy Server's batch pI computational tool, using the 10 residue peptide sequence centered on the S/T-P motif of each site as a representative model (Gasteiger et al., 2005).

## Results and discussion

### Binding by the PPIase domain phosphate-binding loop is required for Pin1 interaction with select targets

**Figure 2:**
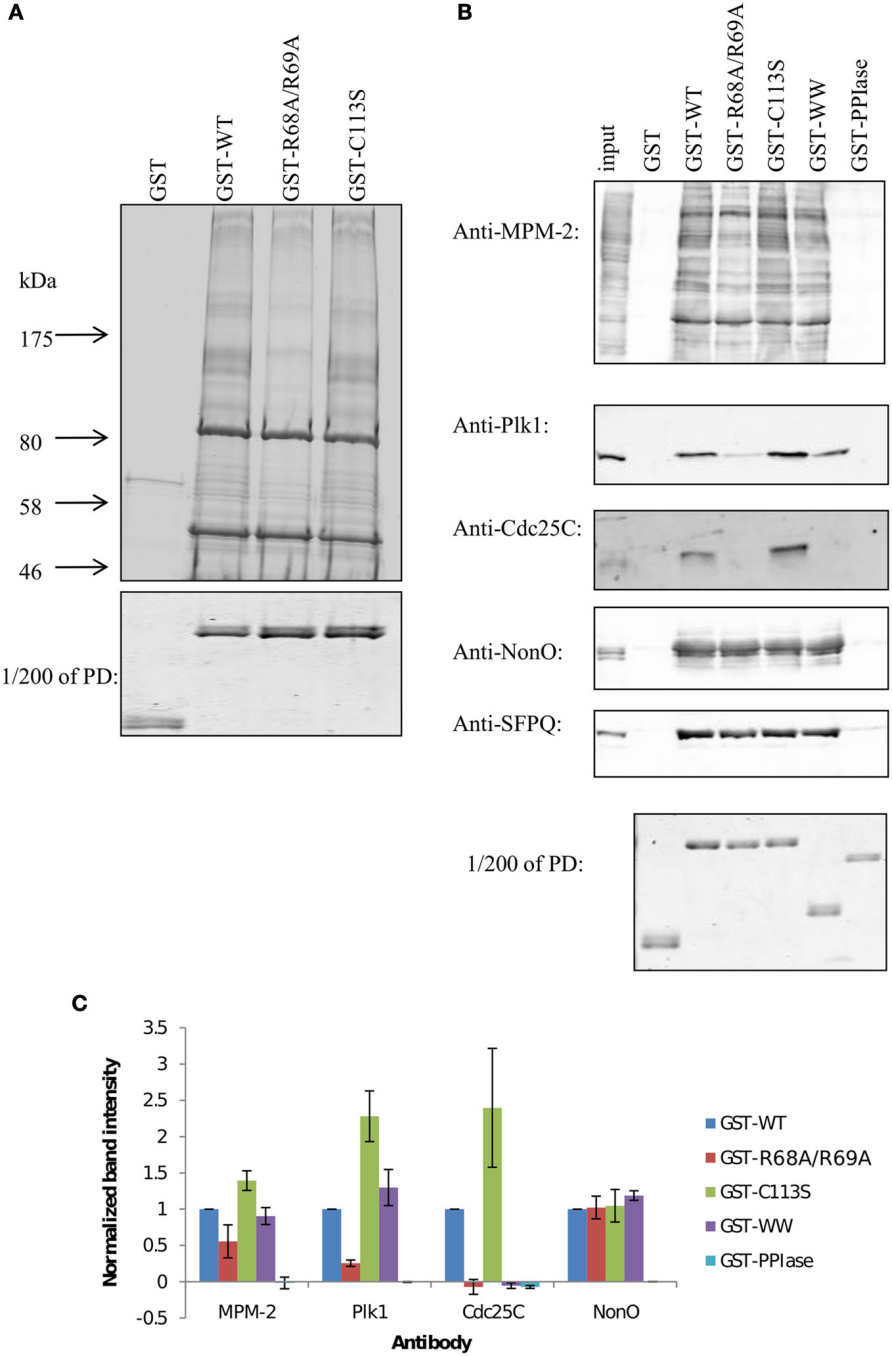
**GST pull-downs with Pin1 mutants reveal two different types of binding proteins. (A)** Large-scale GST pull-downs. One to two milligrams of nocodazole-treated HeLa lysates were incubated with GST fusion proteins bound to glutathione beads. Interacting proteins were run on a 5–12% gradient gel and stained with Coomassie Blue. **(B)** GST pull-downs were performed as in **(A)** with 1 mg of HeLa lysate. Interacting proteins were run on 10% SDS-PAGE, transferred to PVDF and blotted with the indicated antibody. To better compare the amount of fusion protein on the beads, 1/200 of each pull-down was run on a gel and stained with Coomassie Blue (PD). **(C)** Quantification of blots shown in **(B)**. Results are the mean of three independent pull-down experiments ±S.E.M.

Two catalytically deficient mutants of Pin1 were previously generated, one in which the R68 and R69 residues of the PPIase domain phosphate-binding loop were mutated to alanine (R68A/R69A), and the other in which C113 of the active site was mutated to serine (C113S) (Zhou et al., 2000; Behrsin et al., 2007). To assess the role of the PPIase domain in target binding, we used GST fusion proteins encoding wild-type Pin1 or the catalytically deficient Pin1 mutants to pull down Pin1 interacting proteins in nocodazole-arrested HeLa lysates. Figure 2A shows that wild-type Pin1 (lane 2) and the C113S mutant (lane 4) are equally capable of pulling down Pin1 target proteins. In contrast, the R68A/R69A mutations (lane 3) resulted in an overall decrease in binding. R68 and R69 are part of a large, somewhat flexible loop that coordinates the phosphate when substrates are bound to the PPIase domain (Ranganathan et al., 1997; Verdecia et al., 2000). The fact that the R68A/R69A mutations negatively affected target binding while the C113S mutation did not suggests that the decreased binding seen with R68A/R69A is not related to isomerase activity but is due to a binding defect in the PPIase domain. This result was unexpected, since the reported higher affinity of the WW domain for phosphorylated motifs was thought to mediate Pin1 target binding.

To further dissect the role of the PPIase domain in target binding, GST pull-downs were carried out using the same mitotic extracts, and mitotic phospho-proteins were specifically identified with the MPM-2 antibody (Figure 2B, top panel), an antibody generated against a mitotic lysate that recognizes common Pin1 target phosphoepitopes (Davis et al., 1983; Yaffe et al., 1997; Shen et al., 1998). Again, wild-type Pin1 (GST-WT) and the C113S mutant (GST-C113S) yielded similar profiles. The R68A/R69A (GST-R68A/R69A) and the isolated WW domain (GST-WW) were somewhat compromised in their ability to bind mitotic phospho-proteins; the isolated PPIase domain (GST-PPIase) failed to pull down any phospho-proteins. Thus, the PPIase domain appears to play a role in target binding. This appears to be primarily in conjunction with the WW domain, but we cannot rule out WW domain independent targets. The prediction that the WW domain and PPIase domain have different specificities is further reinforced by the identification of PPIase domain-specific inhibitors that do not interact with the WW domain (Duncan et al., 2011).

These PPIase domain-dependent interactions led us to hypothesize that select multiply phosphorylated targets may bind with high affinity to Pin1 by simultaneous low-affinity interactions with both the WW domain and the PPIase domain. The loss of binding due to the R68A/R69A mutations was not globally affecting Pin1 targets, suggesting that binding of one target population requires only the WW domain, while binding of a second target population requires both the WW domain and the PPIase domain with an intact phosphate binding loop (PBL).

The hypothesis that particular targets require simultaneous interaction with both the WW and the PPIase domain was tested using the known Pin1 targets Plk1 and Cdc25C (Figure 2B, 2nd and 3rd panel, Figure 2C). Both proteins bound well to full-length Pin1, and equally well to the catalytically deficient C113S mutant. Following the trend of some of the proteins from the MPM-2 blot above, Plk1 and Cdc25C bound poorly to the R68A/R69A mutant of Pin1. Consistent again with the MPM-2 blot, the PPIase domain was unable to bind these proteins, whereas the isolated WW domain also bound poorly. This is surprising given that the WW domain and the PPIase domain exhibit respective *K*_D_ values of 1.2 μM and 11 μM to the optimal Pintide substrate (WFYpSPFLE; Lu et al., 1999b). The fact that both the isolated WW domain and R68A/R69A mutant are deficient in binding to Plk1 and Cdc25C—whereas the C113S mutant shows no such deficit—indicate that the PPIase domain plays a role in binding these proteins, and this role can be traced to its phosphate-binding loop. This suggests that in cases where the WW domain has weaker affinity for Pin1 targets, the PPIase domain phosphate-binding loop can contribute, perhaps by binding to a second Pin1 site.

Such a role for the PPIase domain in target binding is not universal. In this respect, the two most prominent bands in our pull-down, located at ~50 kDa and ~85 kDa bound similarly to all GST fusions tested, including GST-R68A/R69A. These bands were identified using MALDI-TOF MS as NonO/p54^nrb^ and its obligate binding partner SFPQ/PSF. The identities of these bands were further confirmed by immunoblotting with antibodies specific to NonO and SFPQ. Both NonO and SFPQ showed robust binding to all GST fusion proteins except the isolated PPIase domain.

**Figure 3:**
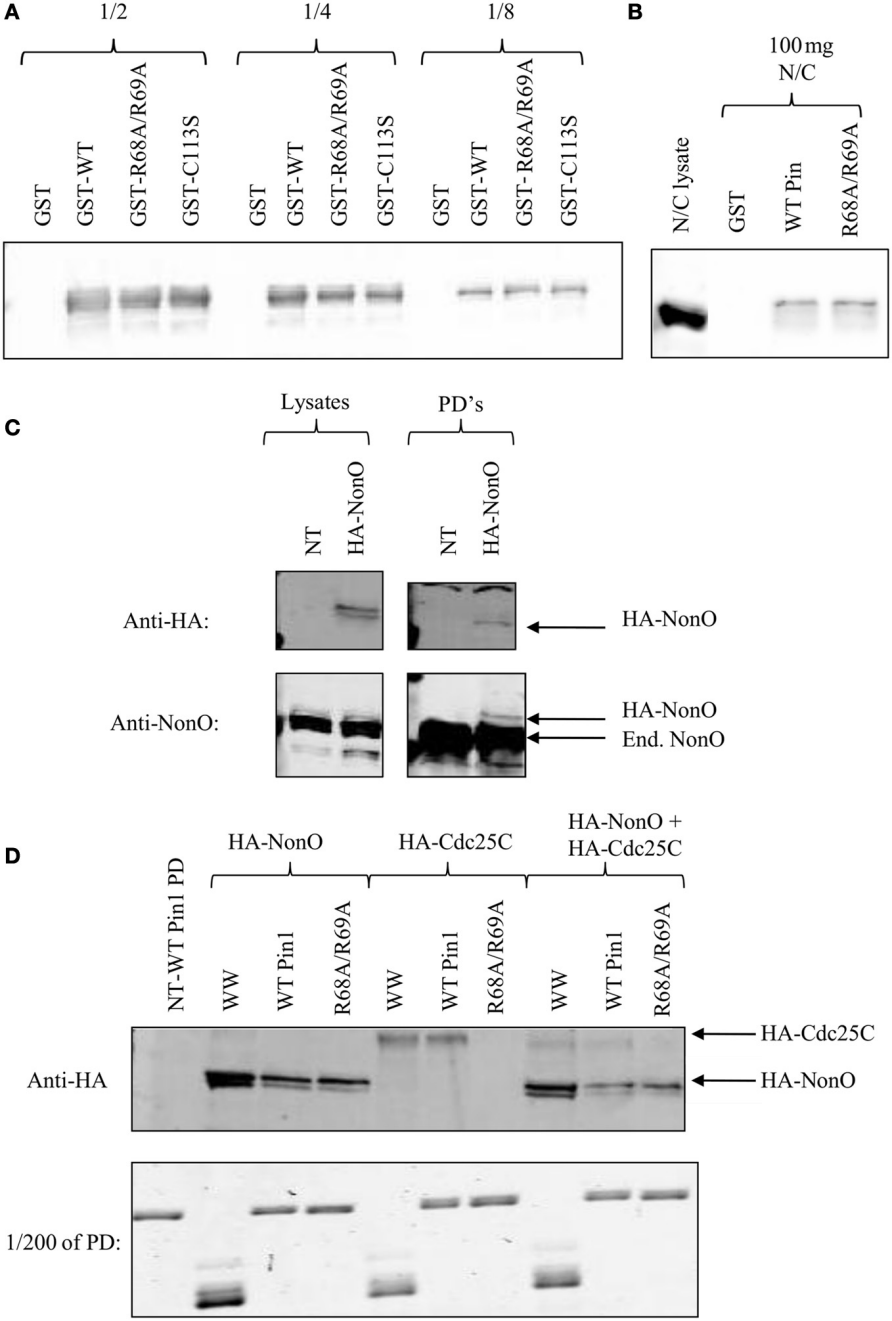
**p54nrb/NonO binding to R68A/R69A is not due to protein abundance. (A)** One to two milligrams of nocodazole-treated HeLa lysates were incubated with GST fusion proteins bound to glutathione beads. Serial dilutions of the pull-down were run on a 5–12% gradient gel, and immunoblotted with anti-p54nrb/NonO. **(B)** Hundred micrograms of nocodazole-treated HeLa lysates (N/C) were incubated with GST fusion proteins bound to glutathione beads. Interacting proteins were run on a 5–12% gradient gel and stained with Coomassie Blue. **(C)** HeLa cells were either transfected with HA-NonO or left untransfected (NT). Lysates were used in GST pull-downs (PD's) as above and immunoblotted with the indicated antibody. Endogenous NonO is indicated with End. **(D)** HeLa cells were transfected with HA-NonO or HA-Cdc25C or left untransfected (NT). Lysates were used in pull-downs (PD) as above and transfected proteins were detected with anti-HA. For the combined sample, 1 mg of HA-NonO lysate was mixed with 1 mg of HA-Cdc25C lysate.

We wanted to ensure that the PPIase domain-independent binding of NonO and SFPQ was not due to their abundance. Therefore, pull-downs with Pin1 mutants were performed and serial dilutions of the pull-down run on a Western Blot. Lower serial dilutions did not show any difference between R68A/R69A and wild-type with respect to their interactions with NonO (Figure 3A). NonO levels were also the same when a lower amount of lysate was used in the pull-downs (Figure 3B). We then exogenously expressed NonO at lower levels than the endogenous protein (Figure 3C) and compared levels of both HA-NonO and HA-Cdc25C in Pin1 pull-downs on the same blot. We found that, like their endogenous counterparts, NonO still bound R68A/R69A while Cdc25C did not (Figure 3D). Collectively, these results confirm that binding of NonO to Pin1-R68A/R69A is not simply due to its abundance.

We have identified two groups of proteins—one that includes NonO and SFPQ, and a second including Cdc25C and Plk1—that demonstrate different sensitivity to the loss of the PPIase domain phosphate-binding loop. The differences in binding suggests that the PPIase domain and the WW domain bind simultaneously to two pS/T-P sites in Cdc25C and Plk1, whereas the WW domain is sufficient for binding to a single pS/T-P site in NonO and SFPQ. Consequently, there may be at least two different classes of Pin1 interactors: one class that requires an intact PPIase domain phosphate-binding loop for their association with Pin1 (which we will refer to as PBL-dependent binding proteins) and another where an intact PPIase domain is not critical for binding (named PBL-independent binding proteins). To our knowledge, this is the first study to suggest that the Pin1 isomerase domain plays a direct and critical role in the association of Pin1 with a subset of its interacting proteins.

### Sequence characteristics of Pin1 binding sites

**Figure 4:**
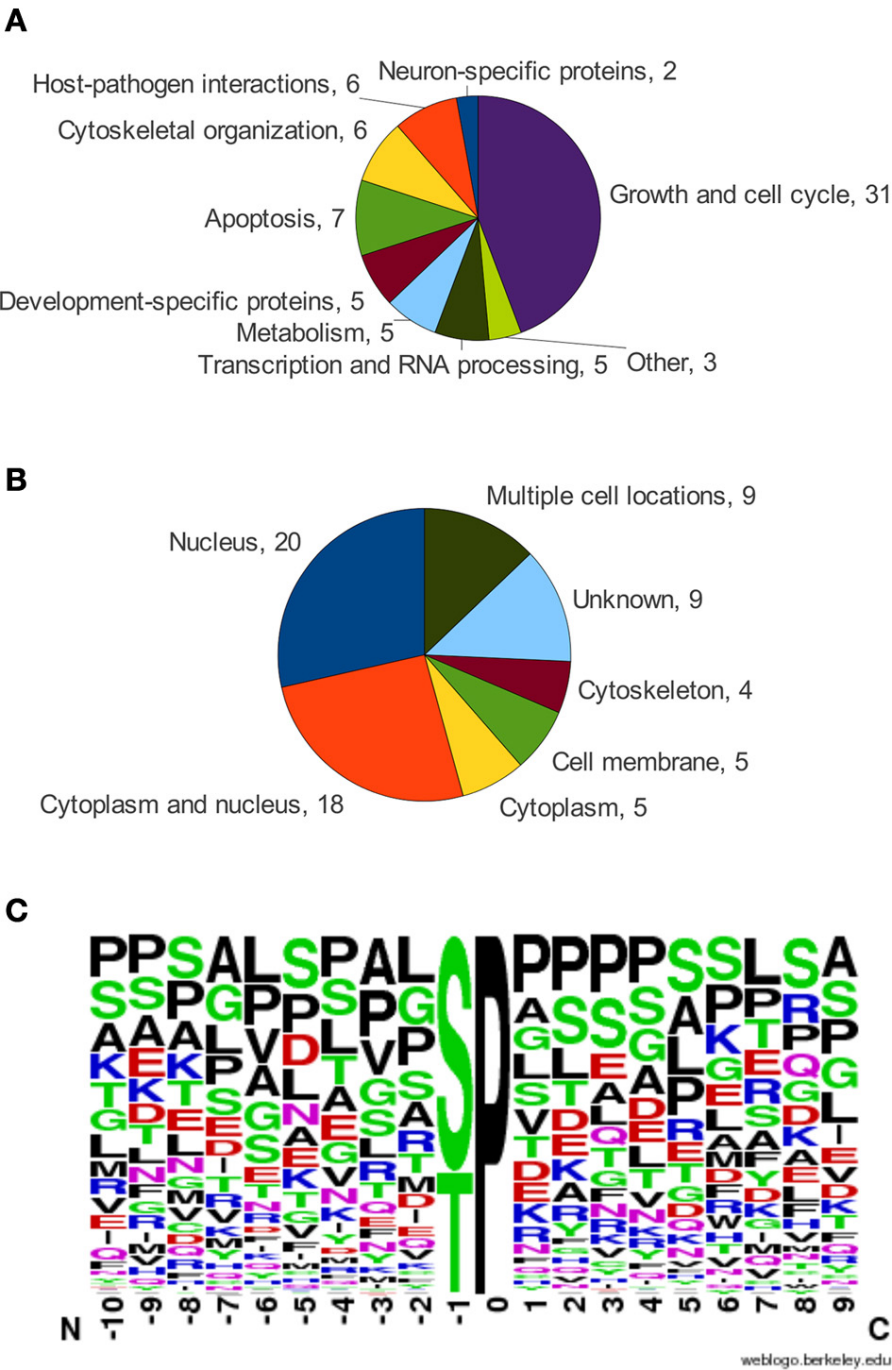
**(A)** Functional categorization of Pin1 interactors. **(B)** Subcellular location of Pin1 interactors, from UniProtKB. **(C)** Frequency chart of amino acids in the alignment of Pin1 binding sites oriented at the S/T-P motif, using the WebLogo service (Schneider and Stephens, 1990; Crooks et al., 2004).

In an effort to identify the determinants for PBL-dependent and -independent binding, we examined the binding site sequences of known Pin1 interacting proteins. Only Pin1 targets that have defined binding sites for Pin1, where mutation or abrogation of the site led to a loss of physical interaction as determined by pull-downs or immunoprecipitations, were used in the analysis. With these criteria, we found 71 proteins containing a total of 120 Pin1 binding sites (Data Sheet 1). Cellular functions of these 71 proteins varied with almost half involved in growth and the cell cycle (Figure 4A). Furthermore, over half of the proteins identified can be found in the nucleus (Figure 4B) consistent with Pin1 localization (Lu et al., 1996). In the 120 sites analyzed, 98 were predicted to be in regions of disorder, and the remaining 22 sites were in surface loops rather than regions of constrained secondary structure. This is consistent with Pin1 preferring to act on flexible, unstructured regions of the protein, which allow space for the 180° rotation needed for isomerization (Lippens et al., 2007). As expected, given the exposed hydrophobic surface of the Pin1 catalytic site, the binding sequences are relatively hydrophobic.

Analysis of the Pin1 binding sites showed considerable variability at positions around the S/T-P motif (Figure 4C). Residues surrounding the pS/T-P motif in Pin1 binding sites are most frequently proline, serine, leucine, and alanine. This result contrasts with the optimal high affinity Pin1 binding site, W/F/Y-F/I-Y/R/F/W-pS-P-R/F/Y/W-L/I, that was identified through screening of a degenerate peptide library (Yaffe et al., 1997). The very strong preference for aromatic residues is not readily apparent in bona-fide Pin1 binding sites. This observation, along with the degenerate nature of the Pin1 binding sequences identified in bona-fide targets, may be explained by the multitude of signaling processes in which Pin1 is involved (see Figure 4A). Since Pin1 binding is phosphate-directed, its binding sites must also act as targets for kinases. With Pin1 involved in multiple signaling pathways, its targets are phosphorylated by a variety of different kinases, each with different sequence specificity (Data Sheet 1). Additionally, Pin1 may share its binding site with the phosphatases that sometimes act on a protein following a Pin1-catalyzed isomerization event. Thus, Pin1 must have flexible sequence requirements in order to coordinate its actions with multiple signaling pathways.

### Binding sites with a proline at the +1 position may preferentially target the Pin1 WW-domain

**Figure 5:**
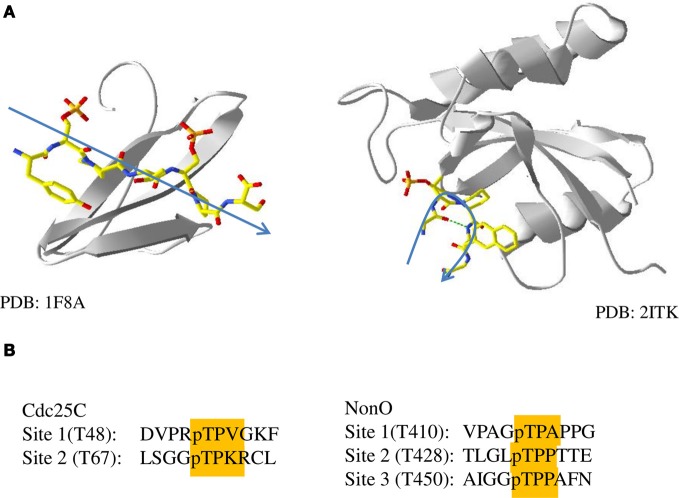
**Phosphopeptide binding by the WW and PPIase domains. (A)** Structures of the WW domain (left, 1F8A) and PPIase domain (right, 2ITK) of Pin1 bound to peptides or peptide inhibitors. A general trace of the backbone of the peptide is shown in blue. **(B)** Sequences of sites in the two Pin1 binding proteins used in this study, Cdc25C and NonO, with pT-P and +1 residues highlighted.

The goal of creating a functional alignment of Pin1 target sequences was to identify differential sequence determinants for the two classes of Pin1 interacting proteins. While this revealed the highly variant nature of the sequence surrounding the canonical pS/T-P binding motif, the minimal information present in the alignment made identifying patterns difficult (Figure 4C). Consequently, we turned to a structural approach to differentiate the two classes of Pin1 targets. Although the two domains of Pin1 bind to the same pS/T-P sequence, structures of these domains with peptides or peptide inhibitors show the bound peptides adopt different conformations. Peptides bound to the WW domain are extended, with little bend induced in the peptide backbone (Figure 5A; Verdecia et al., 2000). The only structures of the PPIase domain bound to a longer peptide are those with a PPIase specific peptide inhibitor (Zhang et al., 2007). The backbone of the peptides in these structures forms a type I β-turn in the active site of Pin1. This places the carbonyl oxygen of the phosphorylated serine or threonine and the amino hydrogen of the amino acid C-terminal to the proline (i.e., in the +1 position) close enough together to form an intramolecular hydrogen bond. However, if the +1 residue in the Pin1 site were proline, then there would not be an amino hydrogen at the +1 position to form this bond. This may alter the affinity of the peptide for the PPIase domain by destabilizing the β-turn conformation that seems to be required for binding. Therefore, pS/T-P sites with an additional prolyl residue at the +1 position (i.e., pS/T-P-P) are unlikely to bind to the PPIase domain.

A +1 proline could explain the difference between PBL-dependent and -independent binding proteins. Interestingly, two of the three Pin1 binding sites in the PBL-independent protein NonO have proline in the +1 position (Figure 5B). Unlike PBL-dependent proteins, which bind both Pin1 domains simultaneously, the two pT-P-P sites in NonO would not bind to the PPIase domain, distinguishing its binding as independent of the PPIase domain. On the other hand, neither of the two sites of Cdc25C contain a proline at the +1 position, suggesting that either may associate with the PPIase domain during binding to Pin1. Unfortunately it is difficult to test the model further, since the binding sites of SFPQ and Plk1 have not been explicitly identified. SFPQ contains two potential Pin1 sites, at pS(33)-P and pT(687)-P (Dephoure et al., 2008), the former of which contains a proline in the +1 position, and therefore fits the model that sites with a +1 proline are associated with PBL-independent binding partners of Pin1. Plk1 also contains two potential Pin1 binding sites at pT(214)-P and pT(498)-P, neither of which contains a +1 proline (Daub et al., 2008). The lack of a +1 proline at either site matches what was seen with the other identified PBL-dependent Pin1 interacting protein, Cdc25C. The presence of a pS/T-P-P binding site may prevent simultaneous binding by both the WW and PPIase domains because the PPIase domain is unable to bind to these sites. In addition, this pS/T-P-P motif may have sufficiently high affinity for the WW domain to allow for the PBL-independent binding seen in the sequential binding model.

There is additional evidence from the literature that pS/T-P-P motifs bind preferentially to the WW domain, and that the presence of these sequences in a target makes its interaction with Pin1 PBL-independent. For example, NMR experiments have shown that the WW domain of Pin1 binds to both pT(212)-P-S and to pT(231)-P-P of Tau, but the PPIase domain only binds to the pT(212)-P-S site, indicating that the +1 proline may confer WW domain-specificity (Smet et al., 2004, 2005). The WW domain-specific nature of the pT(231)-P-P site may also make Tau a PBL-independent Pin1 interactor. This was shown using a K63A mutant of Pin1, which abolishes PPIase domain phosphate-binding by mutating the third critical basic residue of the PBL (Lu et al., 1999a; Behrsin et al., 2007). Phosphorylation of the T(231)-P-P site of Tau is sufficient for interaction with both wild-type Pin1 and the K63A mutant (Lu et al., 1999a), suggesting that Tau is a PBL-independent interactor. Similarly, interaction between K63A Pin1 and peroxisome proliferator-receptor γ (PPARγ) is mediated by pS(84)-P-P (Fujimoto et al., 2010), indicating that PPARγ may also be a PBL-independent target of Pin1. CREB-regulated transcriptional coactivator 2 (CRTC2, aka TORC) also shows PPIase domain-independent binding to Pin1, via the pS(136)-P-P site of its nuclear localization signal (Nakatsu et al., 2010). Thus, we hypothesize that a proline in the +1 position may define a WW domain-specific binding site and have sufficient affinity for the WW domain to allow Pin1 to bind to proteins with this motif independently of the phosphate-binding loop of the PPIase domain.

There are a number of functional implications of this WW domain-specific binding site. In terms of binding models, it would fit both the sequential and multimeric models, as this site would be the initial binding site for Pin1, bringing the PPIase domain proximal to its target sites, whether on the same protein, or other proteins in the complex. However, it may also serve a novel function, through its decreased affinity for the PPIase domain. The behavior of this site in Tau, where the pT(231)-P-P site cannot be isomerized by Pin1, may indicate that these sites cannot be bound and isomerized by the PPIase domain, suggesting that these sites are purely used for protein-protein interaction. Given their high affinity for the Pin1 WW domain, they may serve as reservoirs or sinks, binding Pin1 and sequestering it from its substrates as a regulatory mechanism. It is interesting that almost all Pin1 targets with only a WW domain-specific binding site are found in the cytoplasm, whereas Pin1 and the majority of its substrates are found in the nucleus (Table 1). The cytoplasmic fraction of Pin1 may therefore be predominantly “inactivated” through physical separation from its substrates, by WW domain-mediated association with these proteins. This may have implications when designing therapeutic compounds to inhibit Pin1, since blocking the WW domain would therefore increase the fraction of Pin1 available to its substrates. Further investigation into the various binding models of Pin1 with multiply phosphorylated targets, including WW domain-specific sites, will help clarify the potential functional implications of each binding model.

**Table 1:** **Pin1 substrates containing one or more binding sites with a proline in the +1 position**.

**Name**	**Kinase**	**Effect of Pin1**	**Subcellular location**
FoxO4	Akt	Inhibition	Cytoplasm/Nucleus
Notch1	Erk1/2.JNK	Increased transcriptional activity	Cell membrane
PML	unknown	Destabilization	Cytoplasm/Nucleus
SMAD 3	unknown	Destabilization	Cytoplasm/Nucleus
Myc	GSK-3beta	Dephosphorylation and destabilization	Nucleus
NonO	Cdk1	Unknown	Nucleus
NHERF-1	Cdk1	Dephosphorylation and inhibition	Cytoplasm
PKB	PI3K	Stabilization	Cytoplasm/Nucleus
Tau	unknown	Dephosphorylation and neuroprotective	Cytoskeleton
TFG	unknown	Activation	Unknown
CRTC2	unknown	Inhibition	Cytoplasm/Nucleus
FADK1	Erk1/2	Dephosphorylation and inhibition	Cytoplasm/Nucleus
Mcl-1	Erk1/2	Stabilization	Cytoplasm/Nucleus
NF-kappa-B p65	unknown	Stabilization	Cytoplasm/Nucleus
Oct3/Oct4	unknown	Stabilization and increased transcription	Nucleus
PPAR-gamma	MAPK.JNK	Stabilization	Cytoplasm/Nucleus
RARalpha	Cdk7	Destabilization	Cytoplasm/Nucleus
SF-1	Cdk7.Erk1/2	Increased transcriptional activity	Nucleus
Tax-1	unknown	Activation	Cytoplasm/Nucleus

Highlighted entries contain only the +1 proline binding site.

## Concluding remarks

This study characterized the binding properties of the R68A/R69A mutant of the Pin1 isomerase domain, and in doing so, identified a class of Pin1 interactor that requires both the WW domain and phosphate-binding loop of the PPIase domain for protein–protein interaction. To account for the binding characteristics of these targets, a new simultaneous model of binding was proposed, in which the simultaneous binding of the two domains of Pin1 at two independent sites on an interacting protein overcomes poor binding by each of the individual domains. In an attempt to identify the sequence or structural determinants separating these two classes of interactors, a comprehensive analysis of Pin1 interacting proteins with known binding sites was undertaken. Following a structural comparison of peptides bound to the WW and PPIase domains of Pin1, we proposed that a proline positioned C-terminal to the pS/T-P Pin1 binding site decreases PPIase domain affinity, and the interactions between Pin1 and these targets are therefore mediated exclusively through the WW domain. The concept of a WW-domain-specific binding site helps reinforce the sequential model of Pin1 binding, in which the WW-domain binds one site of a multiply phosphorylated target protein, targeting the catalytic domain to the appropriate site. Further studies comparing singly and multiply phosphorylated Pin1 targets must be undertaken to evaluate both the simultaneous and sequential models of Pin1 interaction. While the importance of Pin1 in oncogenesis is understood, attempts to use it as a therapeutic target may be impeded by the various ways in which Pin1 interacts with its targets. In this regard the identification of two classes of Pin1 interactors is highly relevant to pharmaceutical development, as it indicates the importance of developing agents to each of the individual domains.

### Conflict of interest statement

The authors declare that the research was conducted in the absence of any commercial or financial relationships that could be construed as a potential conflict of interest.

## Research Topic:

From structural to molecular systems biology: experimental and computational approaches to unravel mechanisms of kinase activity regulation in cancer and neurodegeneration

## Topic Editor:

Matteo Barberis, Humboldt University Berlin, Germany; Max Planck Institute for Molecular Genetics, Berlin, Germany
